# Prediction of bruise volume propagation of pear during the storage using soft computing methods

**DOI:** 10.1002/fsn3.1365

**Published:** 2019-12-26

**Authors:** Mahsa Sadat Razavi, Abdollah Golmohammadi, Reza Sedghi, Ali Asghari

**Affiliations:** ^1^ Mechanical Engineering of Biosystems Department of Biosystems Engineering Faculty of Agricultural and Natural Resources University of Mohaghegh Ardabili Ardabil Iran; ^2^ Department of Biosystems Engineering Faculty of Agricultural and Natural Resources University of Mohaghegh Ardabili Ardabil Iran; ^3^ Agricultural Machinery Engineering Department of Biosystems Engineering University of Mohaghegh Ardabili Ardabil Iran; ^4^ Department of Biosystems Engineering Faculty of Water and Soil Engineering Gorgan University of Agricultural Sciences and Natural Resources Gorgan Iran

**Keywords:** adaptive neuro‐fuzzy inference system, artificial neural network, bruise, image processing, magnetic resonance imaging, multiple regression, storage

## Abstract

Bruises occur under both static and dynamic loadings when the imposed stress on fruit goes over the failure stress of the fruit tissue. Bruise damage is the main reason for fruit quality loss. In this study, the potential of artificial neural network (ANN), adaptive neuro‐fuzzy inference system (ANFIS), and multiple regression (MR) techniques to predict bruise volume propagation of pears during the storage time was evaluated. For this purpose, at first, the radius of curvature at loading region was obtained. Samples were divided into five groups and subjected to five force levels. Then, they were kept under storage conditions and at 7‐time intervals after loading tests, bruise volume was calculated using magnetic resonance imaging (MRI) and image processing techniques. Force, storage time, and radius of curvature at loading region were employed as input variables, and bruise volume (BV) was considered as output in the developed models. Multilayer perceptron (MLP) artificial neural network with three layers that includes an input layer (three neurons), two hidden layers (two and nine neurons), and one output layer was used. For the evaluation of models, three criteria (RMSE, VAF, and *R* ^2^) were calculated. ANN and MR gave the highest and lowest correlation between predicted and actual values, respectively. These results indicate that the ANN techniques can be used to predict pear bruising propagation in storage time.

## Graphical abstract

The artificial neural network (ANN), adaptive neuro‐fuzzy inference system (ANFIS), and MR techniques to predict bruise volume propagation of pears during the storage time were evaluated. Models have been constructed based on results obtained from magnetic resonance imaging (MRI) and image processing. ANN techniques can be used to predict pear bruising propagation in storage time.

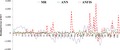

## 1 INTRODUCTION

The pear fruit is a widely consumed product in the world. Improving its quality and appearance is one of the very important issues that the fruit industry is dealing with. Bruising is one of the most common types of postharvest mechanical damage that leads to fruit quality reduction (Knee & Miller, 2002). One of the three types of forces that can cause bruising in pears is the compression load. The loading force is an influential factor in developing pears bruising. Bruising also continues under storage conditions until fruit losses its quality for consumers' consumption. Predicting damage percentage of fruits after harvesting especially during the storage time could lead to better classification of fruits according to their upcoming lost quality. Nondestructive and noninvasive methods are both useful techniques for determining bruises, damages, and many kinds of internal disorders without causing waste of samples. Using these techniques allows us to measure several characteristics simultaneously and also can be used as real‐time methods for bruise detecting, sorting, and classification. These techniques can be applied for both internal and external characterizations such as core breakdown, flesh spot decay, internal browning, senescent scald, watery breakdown, etc. Cameras and machine vision are frequently used for fruit classification according to shape, defects, size, color even on commercial lines. Magnetic resonance imaging (MRI) is one of the nondestructive techniques, which has applied successfully in detecting internal defects. Although this technique is too expensive, while dealing with and using it requires progressive technical knowledge and skills. Constructing models for prediction of bruised volume propagation based on results of magnetic resonance images and image processing techniques can be useful for providing information about the effect of fruit properties on bruise susceptibility and also provide several suggestions for fruit handling. Different statistical and analytical models for the prediction of bruising have been used. Soft computing is a collection of computational techniques in computer science, artificial intelligence, machine learning, and some engineering disciplines, which can be the result of new scientific efforts that make modeling, analysis, and the control of complex systems more easily and successfully. The most important branches of these computational techniques can be considered as fuzzy logic, artificial neural network, and genetic algorithm. An artificial neural network contains informative processes that are able to create and represent a complex correlation between inputs and outputs. The main idea of creating this system is almost inspired by the way that the biological nervous system of the human body works for data processing to learn and producing knowledge. Neural networks, with their striking potential to derive a general solution from intricate or imprecise data, can be applied to take out patterns and detect trends that are too complex to be noticed by either humans or other computer techniques (Simpson, 1990). Artificial neural networks (ANNs) can execute modeling with no assumptions about the nature of the phenomenological mechanisms, and understand the mathematical background of the problem (Fathi, Mohebbi, & Razavi, 2011). In adaptive neuro‐fuzzy inference system (ANFIS), learning abilities of a neural network and reasoning abilities of fuzzy logic are combined to enhance the prediction capabilities of both techniques, as compared to using a single methodology alone. In fuzzy inference system (FIS), each fuzzy rule describes a local behavior of the system. The network structure that performs the FIS and takes on hybrid‐learning rules to train is named ANFIS (Yilmaz & Yuksek, 2009).

## 2 LITERATURE REVIEW

Bruise volumes were determined using mathematical estimation for pear fruits subjected to loading–unloading tests to categorize them based on their susceptibility to bruising (Blahovec & Mares, 2003). Several varieties of pear fruit were tested in the loading–unloading compression test to determine their vulnerability to bruising. The bruise regions were cut and their volumes were determined from the observed cross‐section (Blahovec & Paprstein, 2005). Statistical methods were used to estimate the bruise volume of apple fruits. The regression model predicted the bruise volume with a correlation coefficient of 0.97 (Ahmadi, Ghassemzadeh, Sadeghi, Moghaddam, & Zarif Neshat, 2010). Artificial neural network technique and statistical methods were applied to approximated bruise volume of apple fruits. They constructed the models based on several main independent variables including fruit curvature radius, impact force, impact energy, temperature, and acoustical stiffness. Trial and error approach was applied on the accessible data to select optimal parameters for the network. In that research, ANN model and regression method were used to predict the bruise volume. It was concluded that the ANN is a potential tool for estimating the bruise volume of apple fruits in comparison to the regression model (Zarifneshat et al., 2012). Bruise damage to apple was predicted using the artificial neural network. Their results showed that the model with instruction of five inputs, seven hidden layers, and one output (5‐7‐1), the sigmoid transfer function in the hidden layer and linear transfer function in output layer with 40,000 epochs gives the best correlation between actual and predicted values. These results indicated that the ANN technique could be used to estimate apple bruising in the transport conditions (Rostampour et al., 2013). Bruise volume was used as an index of bruise damage of apple fruit. RBF (radial basis function) artificial neural network and regression models were applied for the estimation of bruise volume. Parameters were determined using trial and error procedures on all data. Their results showed the potential of ANN models for predicting the bruise volume (Zarifneshat, Rohani, Ettefagh, & Saeidirad, 2013). The ability of the ANN technique was assessed as a substitute method for the Maxwell model to estimate the viscoelastic behavior of pomegranate. Neural stress relaxation models were built to describe the stress relaxation behavior of pomegranate concerning time. The neural models were constructed based upon relaxation time and stress relaxation as input and output networks, respectively. The results disclosed that the ANN model has a high ability to provide correct and dependable predictions for stress (Saeidirad, Rohani, & Zarifneshat, 2013). An adaptive neural‐fuzzy inference system model was introduced to detect bruises on Chinese bayberries as a function of the fractal dimension (FD) and RGB intensity values. The ANFIS model with different types of input membership functions (MFs) was developed. The results indicated that for investigating defect, “gauss2mf” MF operated much better than other mentioned MFs. The total correct classification rate of the ANFIS was 90.00%. Thus, the study showed the feasibility of developing a beneficial classification tool for detecting bruises using the ANFIS technique (Zheng, Jiang, & Lu, 2012).

Artificial neural network was applied to develop a classifier system for apple fruits. They used nondestructive method (computer vision system and weight machine, both connected together) to obtain parameters of each fruit (color, damage, size, and weight), which were necessary for modeling. They used ANN for classification of apples using obtained parameters information. Their results showed a low level of error in prediction which verified that the ANN model is effective in estimating apple quality (Bhat, Pant, & Singh, 2014). Determining and predicting of peach fruits injury during the cold storage was investigated using hyperspectral reflectance imaging and ANN method. They applied an multilayer perceptron (MLP) ANN model for reducing data volume obtained by imaging. Also, a model according to the eight selected wavelengths was built for discrimination of cold injury between intact and defected fruits based on quality parameters such as firmness, soluble solid content, titration acidity, chlorophyll content an extractable juice in peaches. Its classification accuracy was 94% for testing samples and 97% for training samples. In their model for predicting chill damage, optimal wavelengths were chosen as inputs for ANN model. The final classification accuracy of chill damage for all samples which were kept under cold storage was 95.8%. Also, predicting the quality parameters using the ANN model had correlation coefficient from 0.69 to 0.90 (Pan et al., 2015).

“DarGazi” variety of Pear fruit is very sensitive to bruising from mechanical impact and compression. Detailed information about estimation models of bruise volume propagation for pear is limited. This study follows our previously published research on pear susceptibility to quasi‐static loadings and estimating its bruised volume using MRI and image processing techniques. This study aims to use ANN and ANFIS for prediction of BV (bruise volume) propagation of “Dargazi” pear based on its radius of curvature in loading region, storage time, and applied force. The obtained results were compared with the traditional statistical model of multiple regression (MR).

## 3 MATERIALS AND METHODS

### 3.1 Experimental details

As it has been reported in our published previous article, the pears used in this study were “Dargazi” variety (*Pyrus communis*). Intact samples without any defects signs were harvested in their physiological ripeness stage (yellowish) from a local garden and for subsequent measurement were transferred to a laboratory, then some of the common physical properties of samples were measured (mass, volume, density, geometrical dimensions, radius of curvature).

#### 3.1.1 Radius of curvature

For measuring the radius of curvature in the location of impact on samples, image processing techniques on RGB obtained images of samples were applied. A wooden box was constructed, and its inside was covered using black sheets to avoid light reflection and provide a uniform imaging condition for all samples. Three fluorescent lamps were mounted triangularly around the camera position above the box. The Canon Powershot G10 camera was used for imaging as connected to a laptop using a USB port to control imaging. Image capturing using PSRemote software was done from 20 × 10^–2^ m distance above of samples. For scaling images, a cubic shape with determined dimensions was placed in position of sample and pictured from the same distance.

#### 3.1.2 Quasi‐static test

After measuring parameters, for simulating the quasi‐static loading, the loading–unloading mechanical test was considered. All samples were divided into five groups randomly and were imposed under the test, each group for a specific range of loads (Figure S1).

#### 3.1.3 Magnetic resonance imaging

For this purpose, samples were placed in a rectangle wooden box where fruits had specific places for sitting on and keeping steady during the imaging. MR images of whole pears were acquired on a Magnetom Symphony 1.5 T system (Siemens), at Kowsar Medical Center. Positioning of samples inside the tunnel of the MRI system and adjusting the image acquisition parameters such as field of view (FOV) and orientations (axial, coronal, and sagittal) are shown in Figure S2. Twenty‐four slices of samples with 0.3‐mm slice gab (slice to slice distance) were captured in coronal orientation (Figure S3) to obtain accurate measuring of bruise volume (Razavi, Asghari, Azadbakht, & Sh, 2018).

#### 3.1.4 Image processing

##### Radius of curvature

ImageJ Software (v. 1.48) was used to measure the radius of curvature. Fitting a circle to at least three points on the fruit surface could give us the radius of curvature, same as a device which is used for measuring curvature. But we considered more points for fitting a circle to get more accurate results.

##### Bruise volume determination using MRI

The bruise volume of samples was measured using image processing techniques over magnetic resonance imaging (MRI) captured of samples. ImageJ software was used to determine bruise volume from the images.

### 3.2 Inputs and output of models

Due to the effect of the fruit properties in the effective forces and fruit vulnerability, the effect of measured physical properties on pear was investigated. The major storage condition parameters that have a significant effect on pear damage were identified, in which was radius of curvature and considered as an independent variable in creating prediction models (results not shown).

Bruise estimation models use the compressive force and time interval as independent variables along with bruise volume. Independent variables used in the regression model, inputs of neural network or ANFIS, consist of imposed force (F) (N), the radius of curvature at loading region (R) (m), storage time (day). The applied loading–unloading force levels were chosen based on previous researches on quasi‐static loading for pear (Blahovec, Vlckova, & Paprstein, 2002). The lowest limit of applied force was based on the applied force during harvesting and sorting; the highest compression level was in pear mechanical handling, transporting, and storage.

### 3.3 Performance evaluation criteria

In this study, three criteria were used to evaluate the models. To evaluate the prediction capability of developed predictive models in the study, “root mean square error” (RMSE), “values account for” (VAF), and the coefficient of determination of the linear regression (*R* ^2^) were calculated, as employed by Yilmaz and Yuksek (2008, 2009), Zarifneshat et al. (2012), Vijayaraghavan et al. (2014), Garg, Vijayaraghavan, Siu Lee Lam, Singru, and Gao (2015), Vijayaraghavan, Garg, Gao, Vijayaraghavan, and Lu (2016) and Vijayaraghavan, Garg, Tai, and Gao (2016). A model is considered as the best when has the smallest RMSE and the largest VAF and *R* ^2^.

### 3.4 Data preprocessing

All data were first normalized over the range of [0, 1]. Every value of a variable *x* was transformed as follows:

(1) $$x_{\text{norm}}=\frac{x-x_{\min}}{x_{\max}-x_{\min}}$$

where *x* is the original data, *x*_norm_ is the normalized values, *x*_max_ and *x*_min_, are the maximum and minimum values of the observed variable, respectively (Anonymous, 2008).

### 3.5 Multiple regression (MR) models

Sometimes two or more variables have a significant effect on the dependent variable. In this case, multiple regression is applied to predict the dependent variable. So the overall goal of MR is to learn more about the relationships among several independent variables and a dependent variable.

### 3.6 Artificial neural network (ANN) models

Perceptron neural networks are considered as feedforward neural networks. Single‐layer perceptron just can classify single‐linear problems, and for more complex problems, it is necessary to use more layers. Multilayer perceptron (MLP) is one of the most widely used neural network architectures for classification or regression problems (Cohen & Intrator, 2002, 2003; Kenneth, Wernter, & MacInyre, 2001; Lim, Loh, Tim, & Shih, 2000). MLP networks consist of an input layer, one or more hidden layers, and an output layer.

In this study, first, the data were split into three subsets: a training set (about 2/4 of all data, 52%), a test set (1/4 of all data, 24%), and a check set (1/4 of all data, 24%). There is no reasonable generalized rule to specify the size of training data for a proper training; nevertheless, the training sample should include all ranges of the available data (Rohani, Abbaspour‐Fard, & Abdolahpour, 2011). The training set can be altered if the operation of the model does not satisfy the expectations (Zhang & Fuh, 1998).

In this study, for the neural network (MLP architecture) analyzing, MATLAB 2015b software was used. Often, when the number of neurons is low in hidden layer, fails to validate the connection of input and output factors. Similarly, when the number of neurons in the hidden layer is high, it causes overfitting (Molga, 2003).

**Figure 1:**
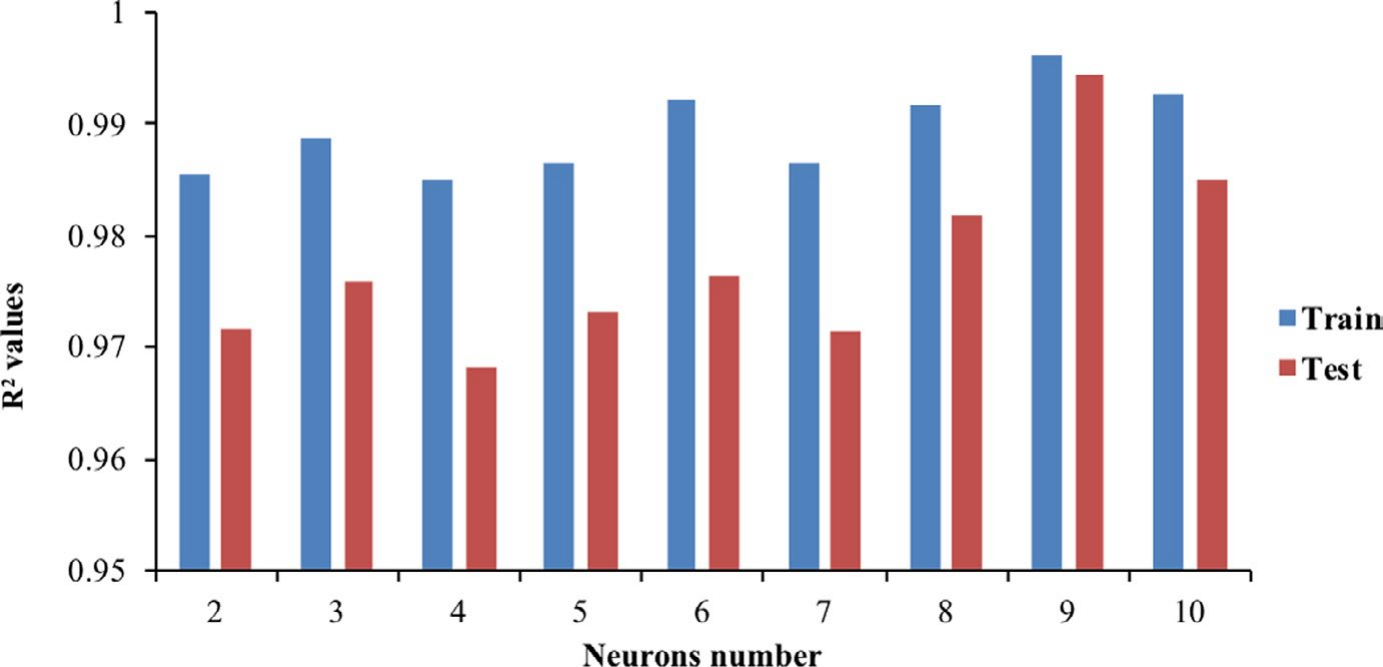
*R* ^2^ values for different number of neurons in second hidden layer and fixed 2 neurons in first hidden layer

**Figure 2:**
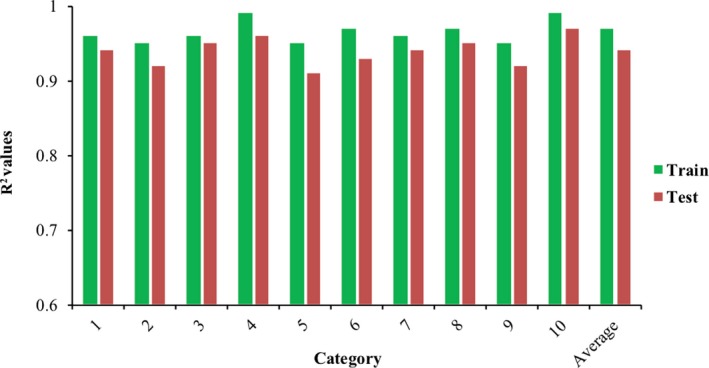
*R* ^2^ values for 10‐fold cross‐validation method

The model had a four‐layer feedforward network that includes an input layer (three neurons), two hidden layers (two and nine neurons), and one output layer. The number of neurons in hidden layers was chosen from a sequence of trial runs of the networks which launched by low number of neurons (two neurons) and enlarged (up to 10 neurons) to get the optimum neurons number in the network. To determine the best topology for the ANN network, *R* ^2^ values were examined as a criterion, and a model which had the highest *R* ^2^ not only for the test data but also for the train data were achieved and selected. Figure 1 shows that the model at neuron number of 2 in the first hidden layer and 9 neurons in the second hidden layer had the closest *R* ^2^ value to 1 which means the strength of the model. Hence, a topology with 2 and 9 neurons in hidden layers was chosen. For the analyzing, network parameters were modified as follows: momentum parameter: 0.3, learning rate parameter: 0.003, networks training function: Levenberg–Marquardt (trainlm), activation (transfer) function for hidden layers: “tansig,” and linear transfer function: “purelin” for the output layer. Levenberg–Marquardt (trainlm) was applied as a training function because this algorithm appears to be the fastest method for training moderate‐sized feedforward neural networks (up to several hundred weights). It also has a very efficient MATLAB implementation, since the solution of the matrix equation is a built‐in function, so its attributes become even more pronounced in a MATLAB setting. The hyperbolic tangent sigmoid (tansig) was employed as a transfer function for hidden layers because it is an excellent choice for nonlinear functions. The MLP neural network structure used in the study is shown in Figure S4. In the end, the K‐Fold cross‐validation method was applied to examine the generalization of the final model. *K* was considered as 10 to divide the data set to 10 categories of features and corresponded targets, and randomly 10–11 of data were selected for each category as train data (about 90% of data were inspected as train data) and the rest (about 10% of data) were applied as test data. Figure 2 shows *R* ^2^ values for different categories of train and test data and the average *R* ^2^ values. A good generalization of the model was demonstrated by the highest *R* ^2^ value (near to 1). Also, Figure 2 shows low differences between different categories in terms of *R* ^2^ values, which confirm the applied topology (2–9 neurons in first and second hidden layers, respectively) can be employed as a fitness function in optimization procedure.

### 3.7 Adaptive neuro‐fuzzy inference system (ANFIS) models

This system is a combination of neural network and fuzzy logic, so it brings all their abilities together in one system. For example, the combination of fuzzy logic and neural network can dismiss the lack of self‐learning ability of the fuzzy logic. In the fuzzy inference system (FIS), each fuzzy rule describes a local behavior of the system. The network structure that performs the FIS and takes on hybrid‐learning rules to train is named ANFIS. The aim of ANFIS is to find a model or mapping that will correctly associate the input values with the target values.

In this study, for predicting BV, ANFIS was applied with three inputs as independent variables (force, storage time, and the radius of curvature) and one output as a dependent variable (BV). MATLAB v. 2015b was used for training the ANFIS model, and Excel v. 2016 was used for computing the performance evaluation criteria and statistical calculations. Figure S5 shows the ANFIS architecture for this study.

Types of parameters and their values which were used in ANFIS model are shown in Table 1.

**Table 1:** Different parameter types and their values used for training ANFIS

ANFIS parameter type	Value
MF type	Gauss function
Number of MFs	5
Output function	Linear
Number of linear parameters	500
Number of nonlinear parameters	30
Total number of parameters	530
Number of training data pairs	53
Number of checking data pairs	26
Number of testing data pairs	26
Number of fuzzy rules	125

## 4 RESULTS AND DISCUSSION

In this paper, the application of MR, ANN, and ANFIS models, for predicting BV of “Dargazi” pear, was described and compared. In order to predict the relation between the obtained parameters in this study, a simple regression analysis was carried out. The relations between BV with other parameters were analyzed. According to the results of simple regression analyses and ANOVA, volume and mass did not have a significant effect on pear damage but, imposed force, storage time, and radius of curvature had a meaningful effect on bruise volume (results not shown). The models of MR, ANN, and ANFIS for predicting of the BV were then built by these three inputs and one output. The obtained results and their basic test statistics are shown in Table S1.

### 4.1 Multiple regression

Multiple regression analysis was performed to correspond the measured BV to force levels, storage time, and radius of curvature in the loading region (Table S2).

**Figure 3:**
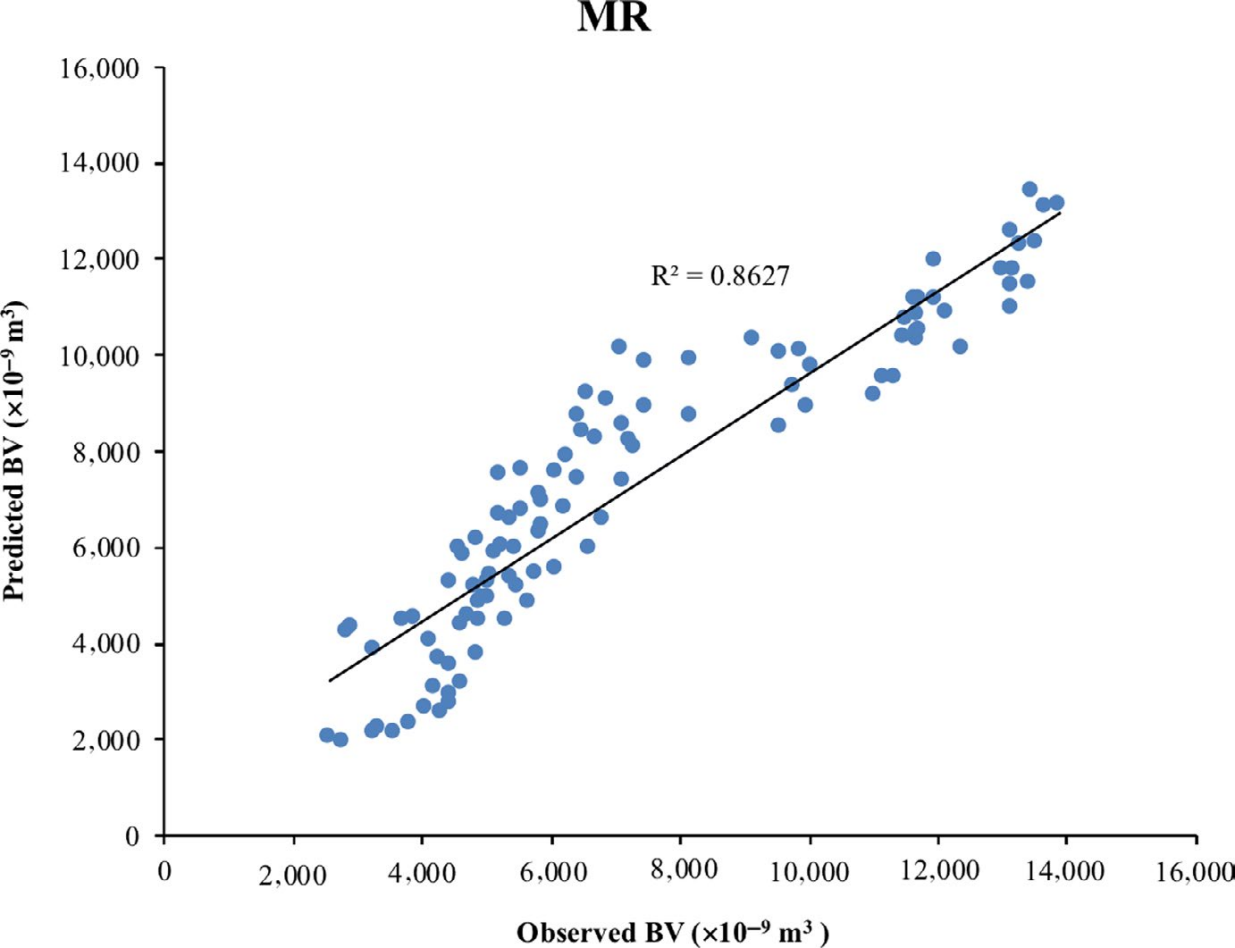
Cross‐correlation of observed and predicted values of BV for MR model

The coefficient of determination between the measured and predicted values is an acceptable index to examine the prediction performance of the model. Figure 3 shows the relationships between measured and predicted values obtained for BV from the MR model. The calculated performance evaluation indices for multiple regression (MR) model are given in Table 2. Ahmadi et al. (2010) applied statistical methods for predicting BV. They built their model by regression method and it could estimate BV with a coefficient of determination (*R* ^2^) of .97. Zarifneshat et al. (2012) predicted BV of apple by regression method with a coefficient of determination (*R* ^2^) of .998. Zarifneshat et al. (2013) used a regression model for predicting bruise volume. In their research, BV could be estimated with a coefficient of determination of 0.969. But, in our research, bruise volume was predicted with a coefficient of determination (*R* ^2^) of .8627. Unlike previous studies on apple fruit, the MR method could not be an accurate model for predicting bruise volume of pear. The accuracy of the analysis for the MR model was 82.53%.

**Table 2:** Performance indices (RMSE, VAF, and *R* ^2^) for MR, ANN, and ANFIS models

Model	Predicted parameter	RMSE	VAF (%)	*R* ^2^
MR	BV	617.05	86.27	.8627
ANN	BV	473.38	99.01	.9909
ANFIS	BV	834.51	91.79	.9336

Abbreviations: ANFIS, adaptive neuro‐fuzzy inference system; ANN, artificial neural network; MR, multiple regression; RMSE, root mean square error, VAF, value account for.

### 4.2 Artificial neural network

**Figure 4:**
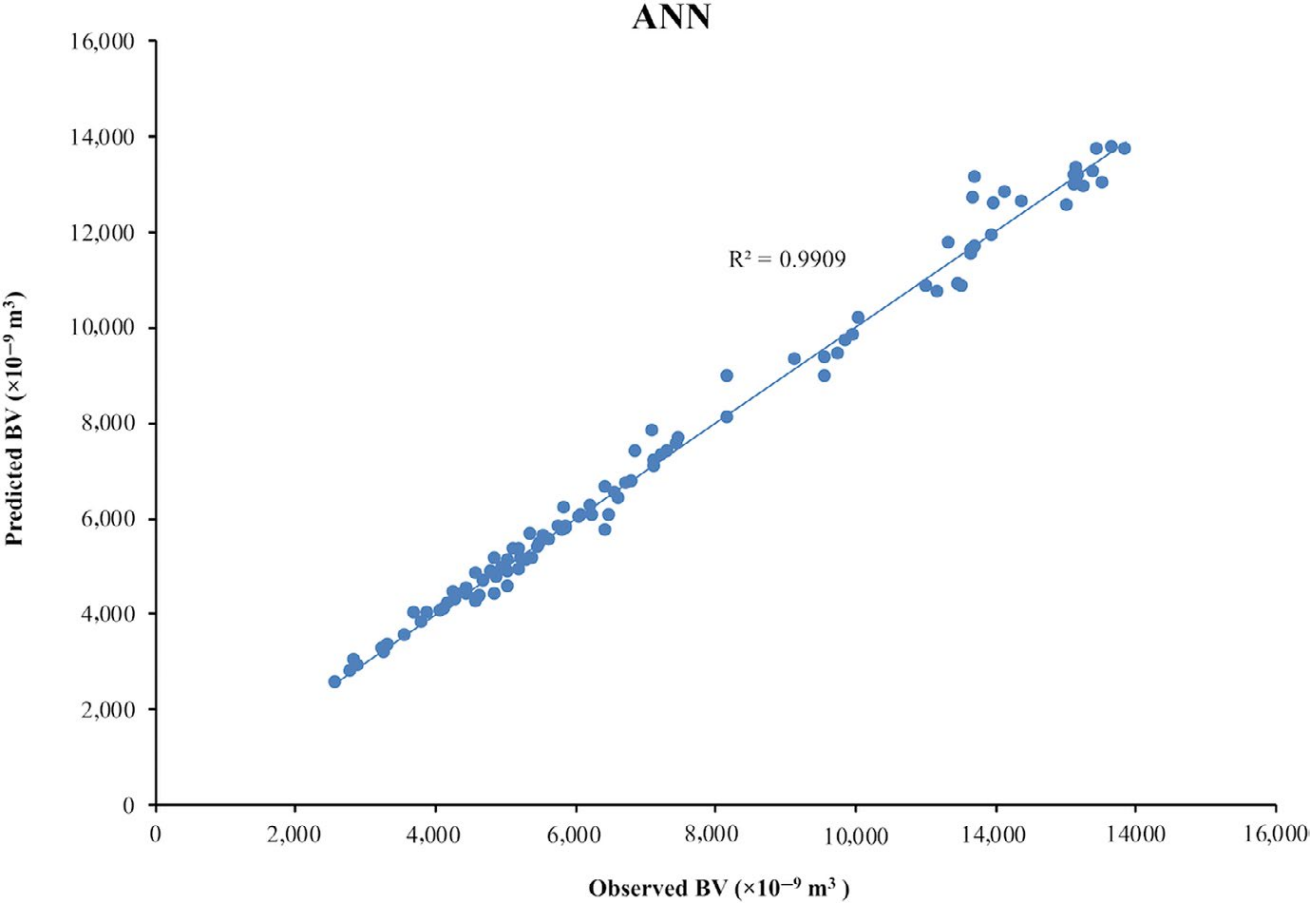
Cross‐correlation of observed and predicted values of BV for ANN model

As seen from Table 2 and Figure 4 of cross‐correlation between observed and predicted values of BV, obtained values of VAF, *R* ^2^, and RMSE demonstrated very high prediction performances. Figure 4 shows the overall coefficient of determination (*R* ^2^) and Figure S6 shows the Pearson correlation coefficients of each phase (training, validation, and test) obtained from the software. The results of training, validation, and test are shown in Figure S6. These results are in agreement with the results of Zarifneshat et al. (2012), Zarifneshat et al. (2013), and Rostampour et al. (2013) researches on bruise damage of apple. In Zarifneshat et al. (2012) research, bruise volume was predicted with a coefficient of determination (*R* ^2^) .978 using the ANN model. Zarifneshat et al. (2012) predicted bruise volume with a coefficient of determination (*R* ^2^) of .998 by the ANN model. Rostampour et al. (2013) predicted bruise volume by ANN model and MLP method with a coefficient of determination (*R* ^2^) of .9998 and 0.9996 in the training and testing phase, respectively. These results indicate the potential of the neural network and verify the obtained results in our research. In our study, BV could be estimated with a coefficient of determination (*R* ^2^) of .9909. Therefore, ANN could be used as a promising and more precise tool for predicting bruise volume. The accuracy of the analysis for the ANN model was 96.28%.

### 4.3 Adaptive neuro‐fuzzy inference system

**Figure 5:**
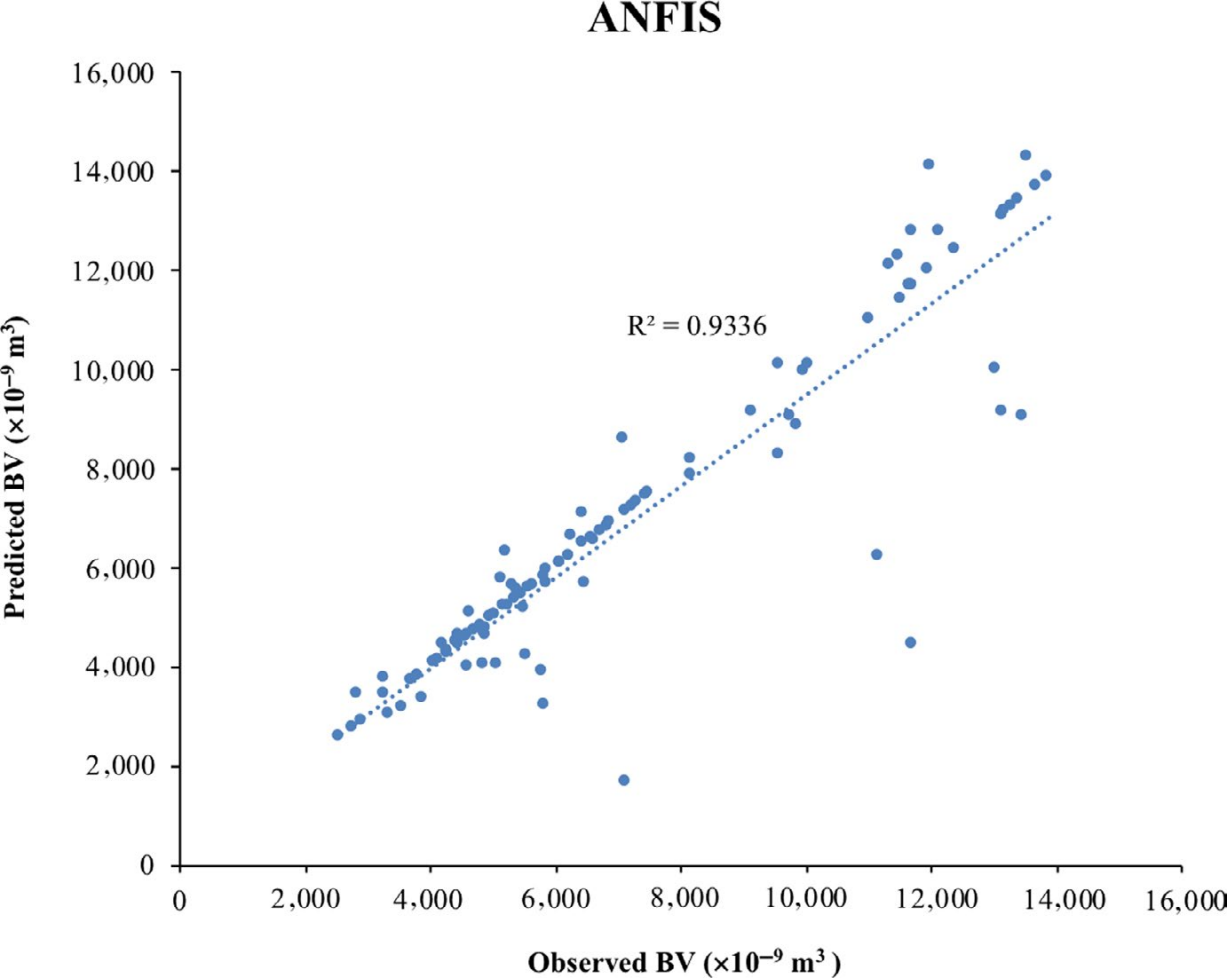
Cross‐correlation of predicted and observed values of BV for ANFIS model

According to the VAF, RMSE, *R* ^2^ values (Table 2), and cross‐correlation between observed and predicted values (Figure 5), constructed ANFIS model for predicting BV has a high prediction performance. This result is in agreement with Zheng et al. (2012) research that the total correct classification rate of the ANFIS was 90.00%. Therefore, these results demonstrated the potential of developing a useful classification tool using the ANFIS technique for detecting bruises. The accuracy of the analysis for the ANN model was 92.4%.

### 4.4 Overall results and comparison of three models

**Figure 6:**
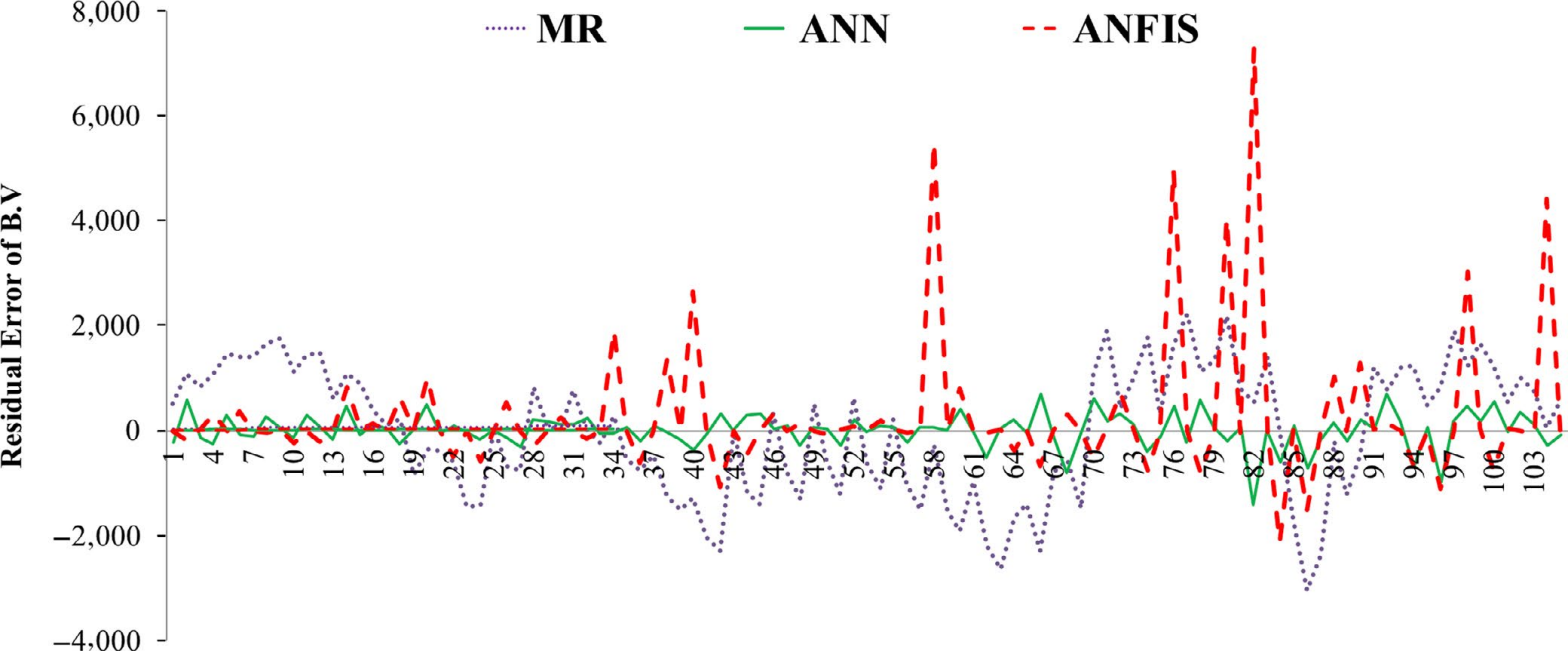
The variation of the values predicted by MR, ANN, and ANFIS model from the observed values

The performance of three models was compared through the statistical criteria of VAF, RMSE, and *R* ^2^. The obtained results as it is shown in Table 2 for prediction of the bruise volume (BV) demonstrated that the MR model has the least prediction performance for prediction of BV according to *R* ^2^ and VAF values, and prediction performance of the ANN model is more accurate than ANFIS and MR according to *R* ^2^ value (which was the highest) and RMSE and VAF values (which were the lowest) and showed the most dependable predictions in comparison with other models. The ANFIS model, for prediction of BV, disclosed a more reliable prediction in comparison with the MR model based on VAF and *R* ^2^ values, but when the RMSE values were considered; the MR model showed better prediction than ANFIS. However, both ANN and ANFIS models matched the predicted and measured values more precisely than the MR model based on *R* ^2^ and VAF values. It can be attributed to this fact that in both models (ANN and ANFIS) a nonlinear relation is applied between input variables, but in the MR model, a linear relation is involved between the variables. In order to show the deviations from the observed values of BV, the distances of the predicted values from the models constructed from the observed values were also calculated; Figure 6 illustrates the mentioned deviation. The graph demonstrated that the deviation interval (−423.2228 to +700.9435) of the predicted values from ANN is less than the deviation interval of ANFIS (−2,125.266 to +7,279.7772) and MR (−3,047 to +2,214). Consequently, ANN can provide the best empirical solution to solve the problem of predicting the pear BV propagation in the storage condition.

## 5 CONCLUSIONS

The previous studies have predicted just bruise volume during the storage condition not its propagation in over time. In our research ANN, ANFIS and MR were used to develop models that can predict the pear bruise volume propagation during storage time. The selection of input variables was the first step to achieve this purpose. The effects of applied force, storage time, and radius of curvature on bruise damage of pear were investigated and meaningful factors were considered as inputs. Also, it was shown that optimum storage time after compression test is about 12 days, and during this time, produces can be consumed by customers, but after that, they would have the lowest quality to be sold. It is shown that the constructed ANN model exhibits more accurate results than the ANFIS model and multiple regression methods for predicting BV. Nevertheless, ANN and ANFIS models may have comparatively similar accuracy. The comparison of their performances showed that a soft computing system is a useful tool for decreasing the ambiguities in the postharvest projects. There is feasibility for predicting BV of pear using the presented experimental relationships and soft computing models. These techniques can be developed and used for online, robust, and automated sorting and grading systems in packing houses or even in gardens for further goals like the estimation of fruits damage percentage that causes them not to be marketable after storage time. To summarize, prediction and estimation of bruise damage volume caused by effective factors of “time, force, the radius of curvature, etc.” in handling, transportation, and storage, and reducing economic losses are an important and necessary process. In this study, ANN represented a more precise and accurate estimation with the lowest error than the other two models. So, it can be introduced and used as an effective and intelligent model for prediction of bruise volume of other strategic produces.

## CONFLICT OF INTEREST

The authors declare that they do not have any conflict of interest.

## ETHICAL STATEMENTS

This material is the authors' own original work, which has not been previously published elsewhere. It is not currently being considered for publication elsewhere. This manuscript reflects the authors' own research and analysis in a truthful and complete manner. The paper properly credits the meaningful contributions of co‐authors and co‐researchers. The results are appropriately placed in the context of prior and existing research. All sources used are properly disclosed. All authors have been personally and actively involved in substantial work leading to the paper and will take public responsibility for its content. Also, this study does not involve any human or animal testing.
