# Supplementary material for: Prediction of bruise volume propagation of pear during the storage using soft computing methods
Source: Food Sci Nutr. 2019 Dec 26;8(2):884–93. doi: 10.1002/fsn3.1365 (PMC7020290; doi:10.1002/fsn3.1365)
Supplement: Supplementary file 1 [file FSN3-8-884-s001.docx]

**Figures**

|  |
| --- |
| **Fig. S1.** Loading-Unloading test |

| 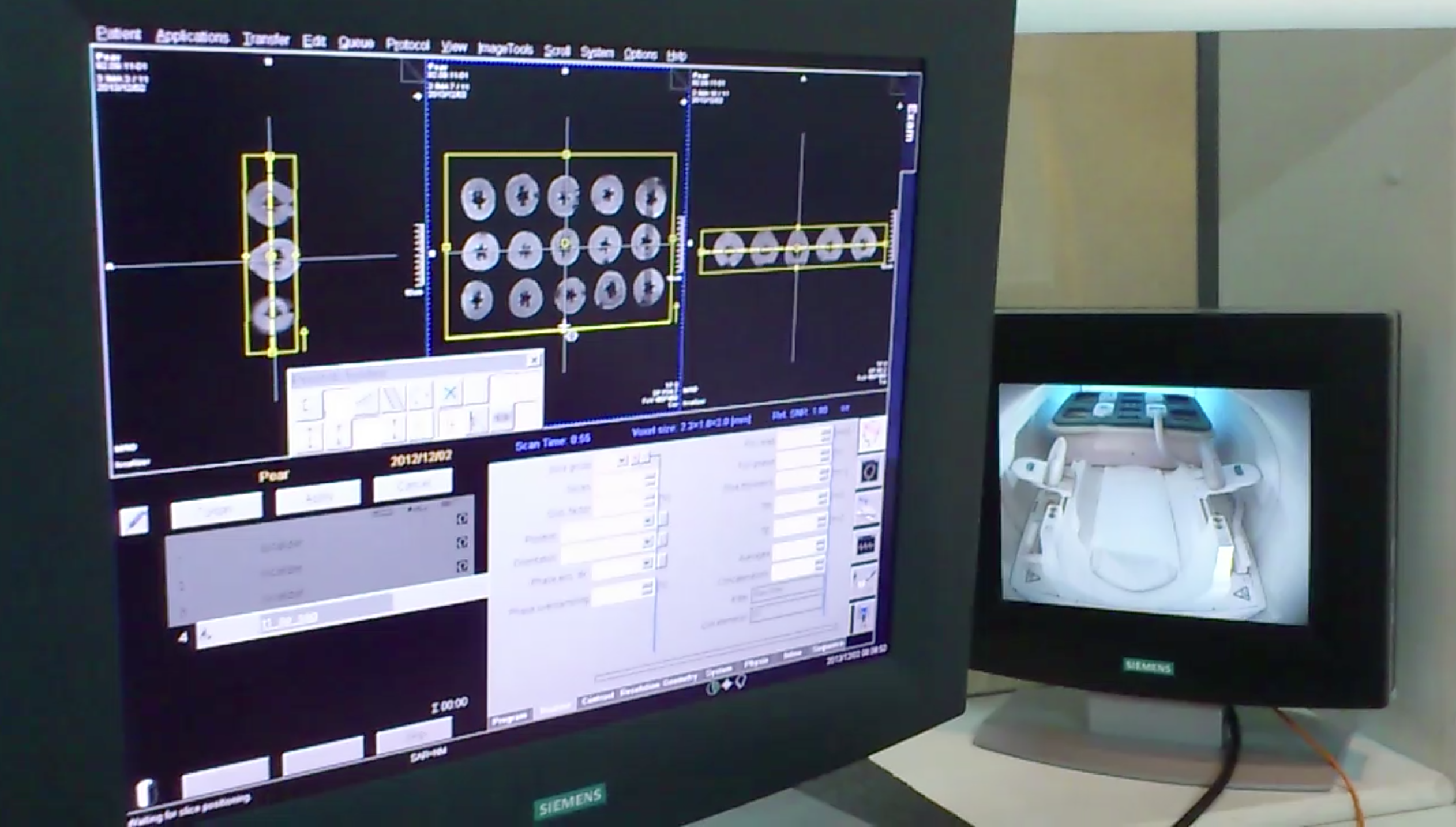 |
| --- |
| **Fig. S2.** Positioning of samples inside the tunnel of MRI system and adjusting the image acquisition parameters |

| 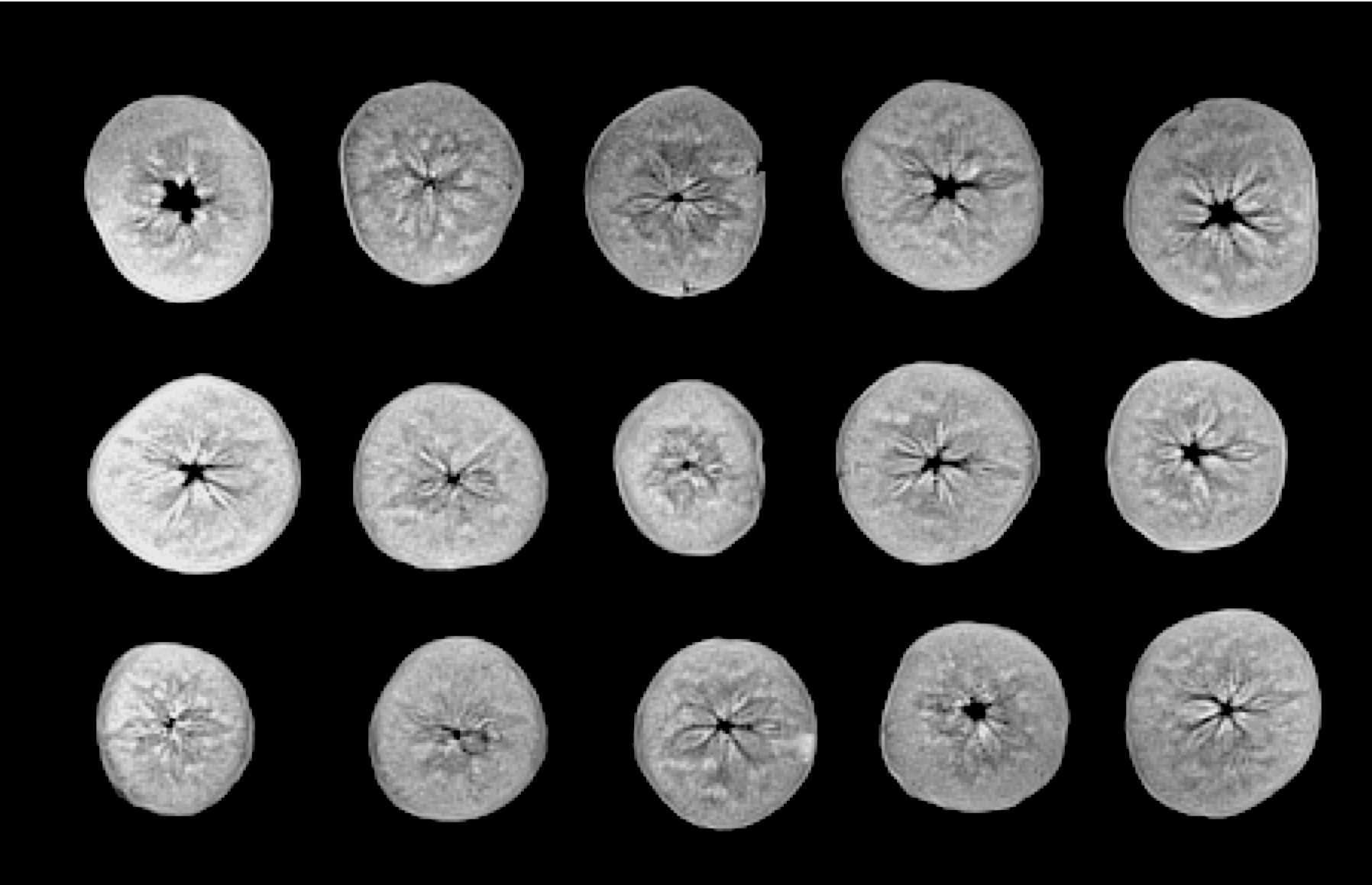 |
| --- |
| **Fig. S3.** One of the Images captured in Coronal orientation |

| 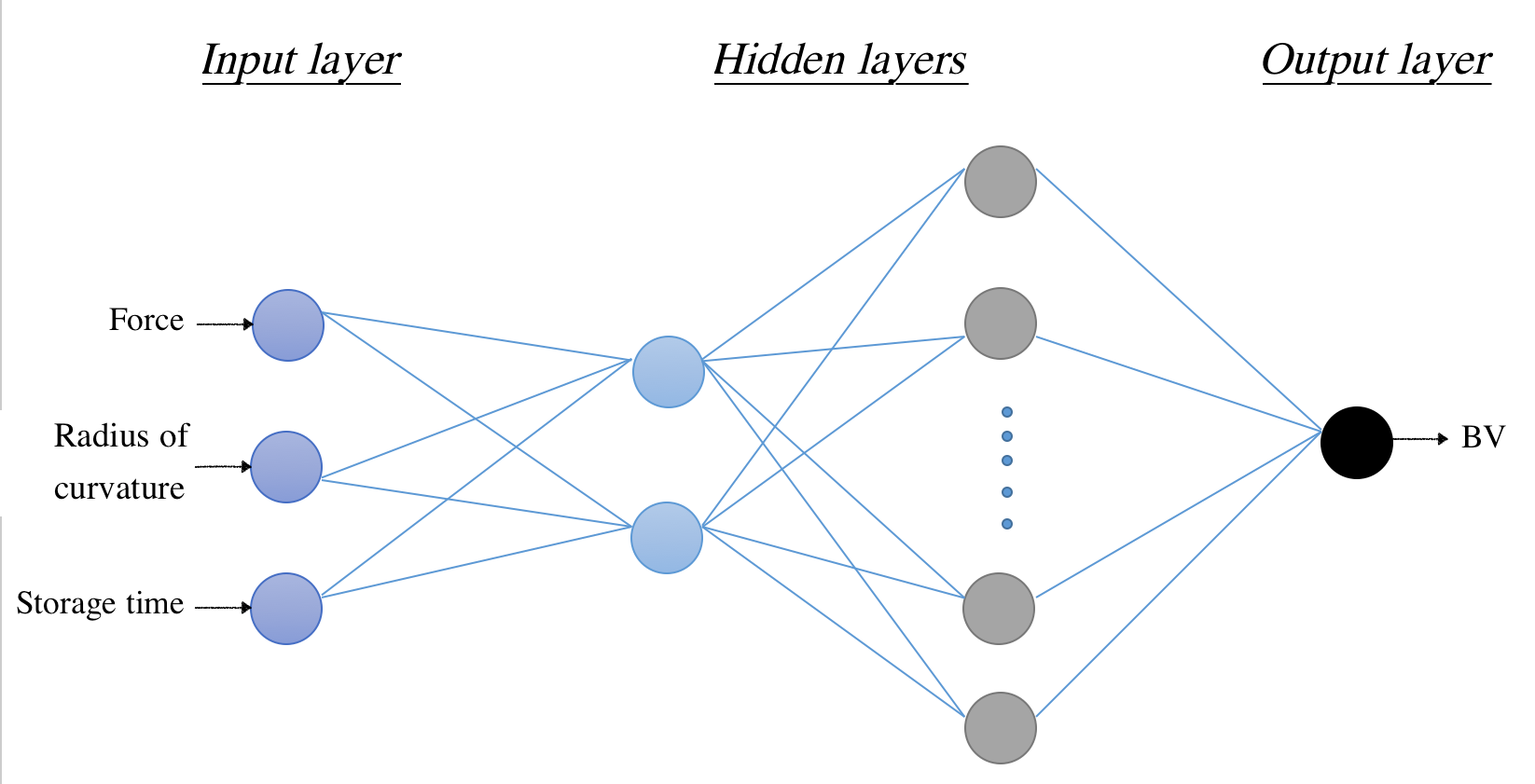 |
| --- |
| **Fig. S4.** MLP neural network structure used in the study |

| 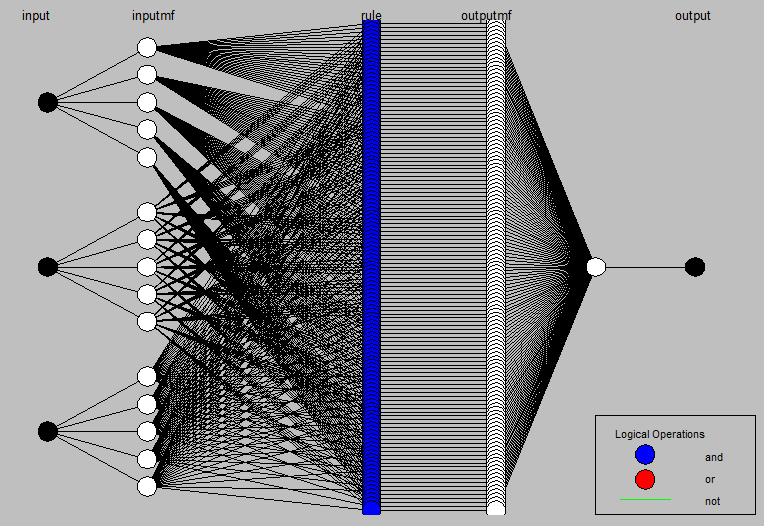 |
| --- |
| **Fig. S5.** ANFIS model architecture for prediction of bruise volume propagation during storage time |

| 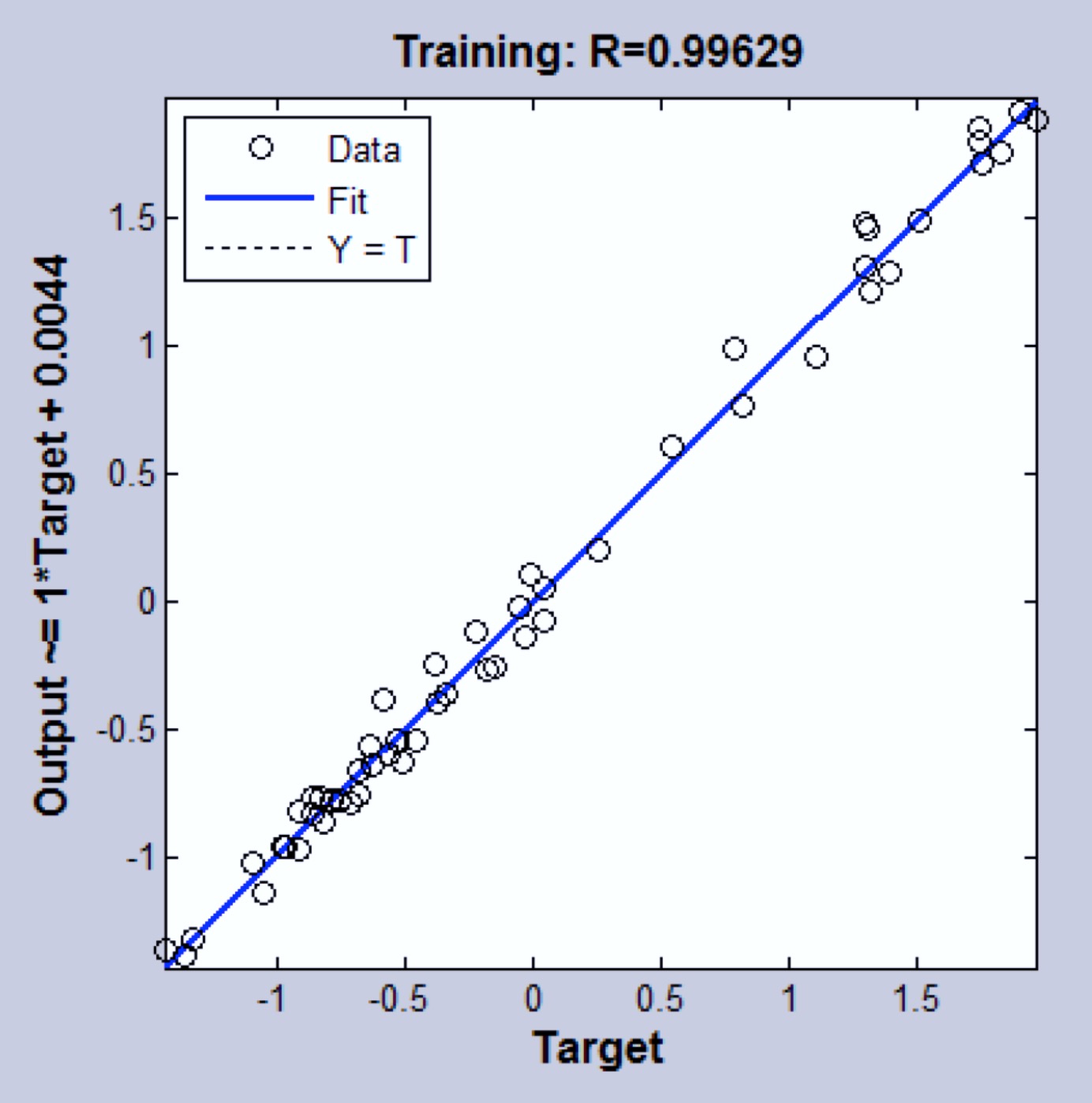 | 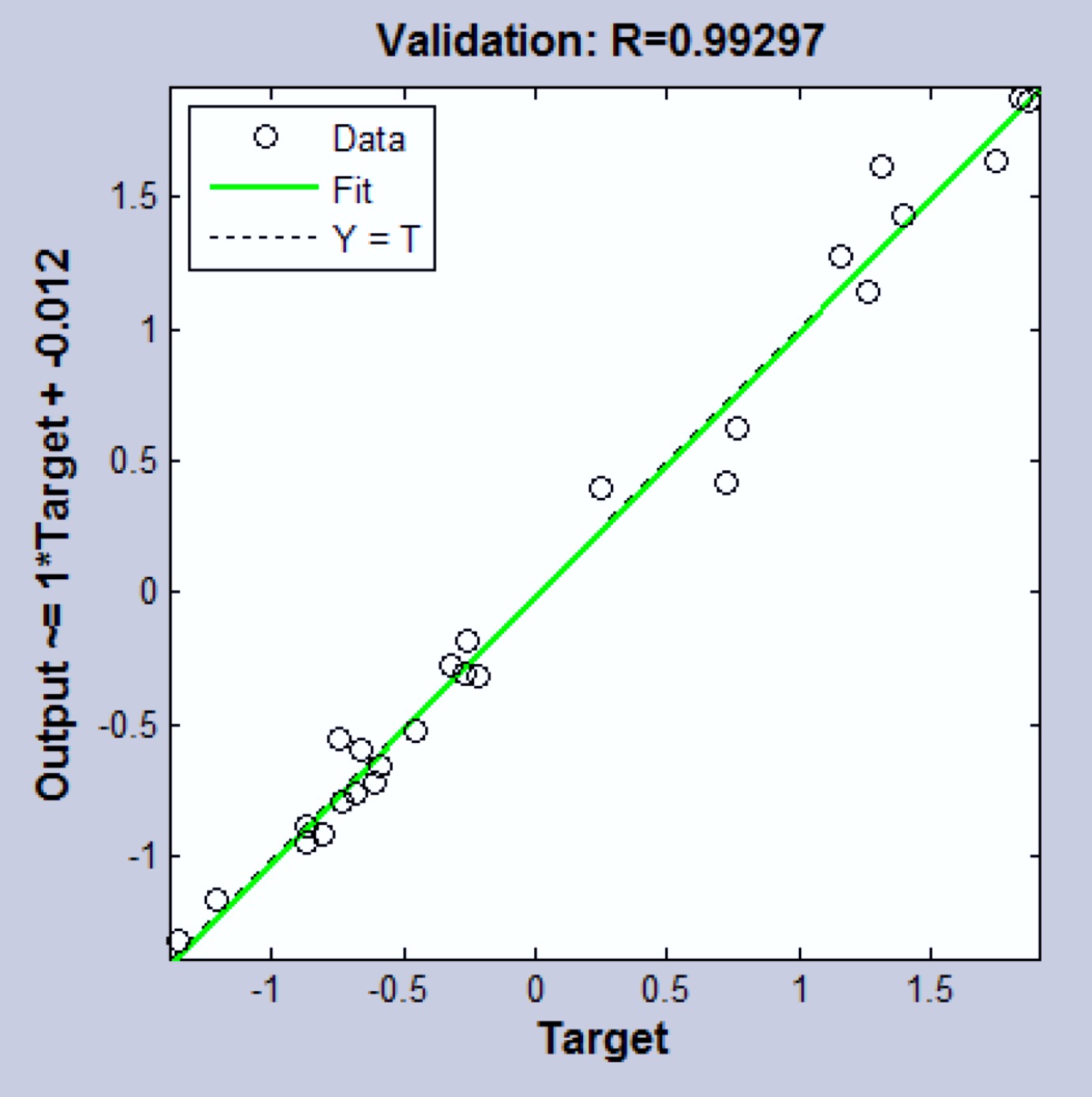 |
| --- | --- |
| 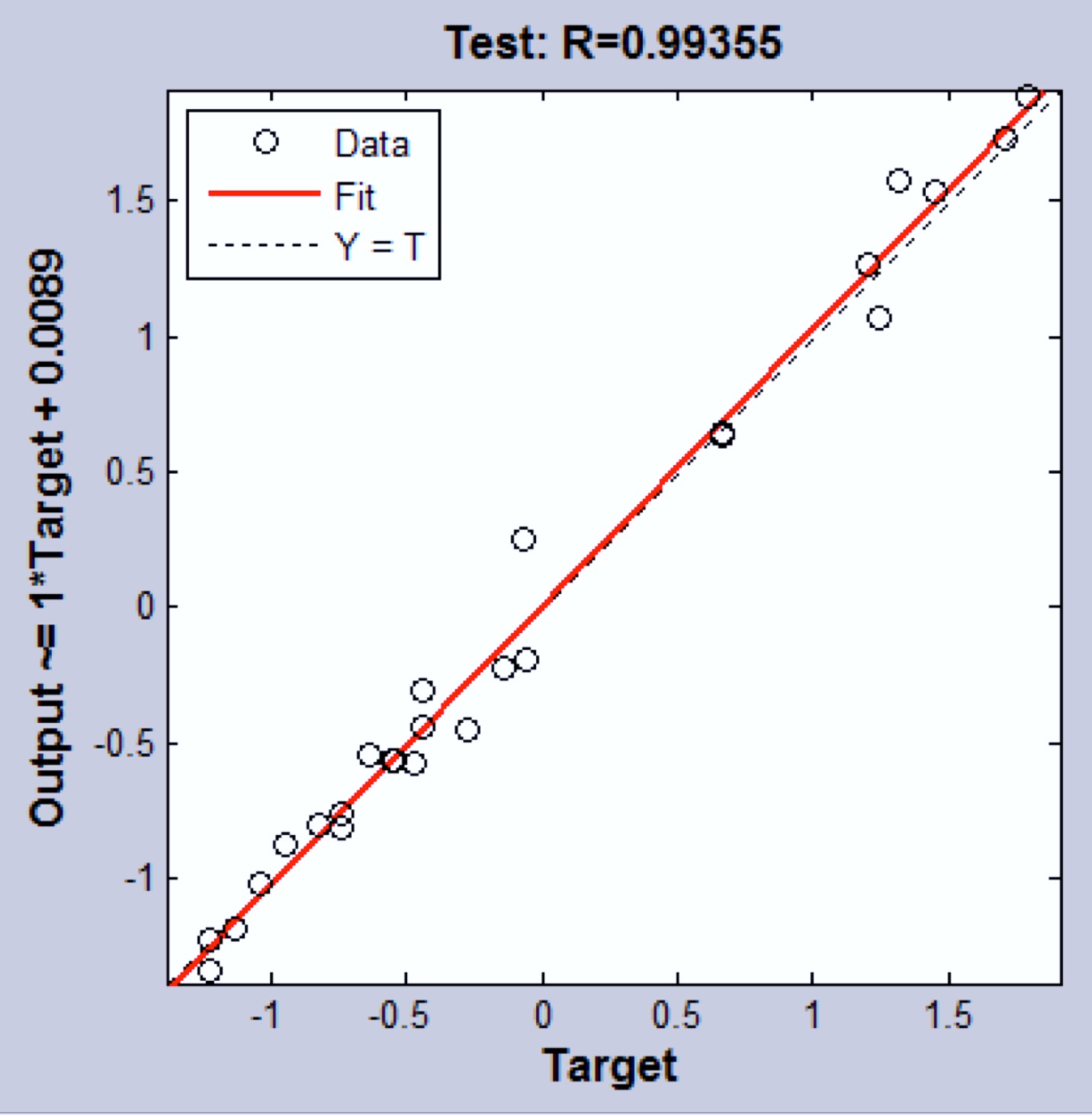 | **Fig. S6.** Results of training, validation and test phases |

**Tables**

| **Table S1** | | | | | |  |
| --- | --- | --- | --- | --- | --- | --- |
| Basic statistics of the results obtained from tests | | | | | |  |
| **Force (N)** | | | | | | **R (mm)** |
|  | 40 | 50 | 60 | 70 | 80 |  |
| **BV (mm3)** | | | | | | |
| **Maximum** | 5056.7551 | 5842.7826 | 7240.2483 | 13180.6909 | **13871.3940** | 43.1860 |
| **Minimum** | **2568.5511** | 2845.1300 | 4586.1958 | 6263.7953 | 7105.7098 | 17.4880 |
| **Average** | 4167.2750 | 4839.7315 | 6009.9603 | 10154.6212 | 11537.1696 | 27.1627 |
| **Std. dev.** | 698.6975 | 969.7435 | 819.3193 | 2206.2482 | 2142.5592 | 5.9628 |
| BV: bruise volume, R: radius of curvature at loading region | | | | | | |

| **Table S2** | | | | | | |
| --- | --- | --- | --- | --- | --- | --- |
| Model summaries of multiple regressions for prediction of BV^*^ | | | | | | |
| Model | | Unstandardized Coefficients | | Standardized Coefficients | t-value | Sig. level |
|  |  | B | Std. error | Beta |  |  |
| 1 | (Constant) | -4751.308 | 721.146 |  | -6.589 | 0.002 |
|  | Force (N) | 206.434 | 8.817 | 0.883 | 23.413 | 0.000 |
|  | Time (day) | 101.506 | 11.133 | 0.336 | 9.118 | 0.000 |
|  | Radius of curvature (mm) | -66.306 | 21.012 | -0.119 | -3.156 | 0.002 |
|  | * Dependent Variable: Bruised volume (mm^3^) | | | | | |
